# Mitochondrial Electron Transport Is the Cellular Target of the Oncology Drug Elesclomol

**DOI:** 10.1371/journal.pone.0029798

**Published:** 2012-01-11

**Authors:** Ronald K. Blackman, Kahlin Cheung-Ong, Marinella Gebbia, David A. Proia, Suqin He, Jane Kepros, Aurelie Jonneaux, Philippe Marchetti, Jerome Kluza, Patricia E. Rao, Yumiko Wada, Guri Giaever, Corey Nislow

**Affiliations:** 1 Synta Pharmaceuticals Corp., Lexington, Massachusetts, United States of America; 2 Donnelly Centre for Cellular and Biomedical Research, University of Toronto, Toronto, Ontario, Canada; 3 UMR 837 – INSERM, Université de Lille II & CHRU LILLE, Lille, France; University of Medicine and Dentistry of New Jersey, United States of America

## Abstract

Elesclomol is a first-in-class investigational drug currently undergoing clinical evaluation as a novel cancer therapeutic. The potent antitumor activity of the compound results from the elevation of reactive oxygen species (ROS) and oxidative stress to levels incompatible with cellular survival. However, the molecular target(s) and mechanism by which elesclomol generates ROS and subsequent cell death were previously undefined. The cellular cytotoxicity of elesclomol in the yeast *S. cerevisiae* appears to occur by a mechanism similar, if not identical, to that in cancer cells. Accordingly, here we used a powerful and validated technology only available in yeast that provides critical insights into the mechanism of action, targets and processes that are disrupted by drug treatment. Using this approach we show that elesclomol does not work through a specific cellular protein target. Instead, it targets a biologically coherent set of processes occurring in the mitochondrion. Specifically, the results indicate that elesclomol, driven by its redox chemistry, interacts with the electron transport chain (ETC) to generate high levels of ROS within the organelle and consequently cell death. Additional experiments in melanoma cells involving drug treatments or cells lacking ETC function confirm that the drug works similarly in human cancer cells. This deeper understanding of elesclomol's mode of action has important implications for the therapeutic application of the drug, including providing a rationale for biomarker-based stratification of patients likely to respond in the clinical setting.

## Introduction

Elesclomol is a novel small molecule drug originally identified in a cell-based screen for its potent proapoptotic activity in cancer cells. More recently, *in vitro* studies indicated that it strongly induces reactive oxygen species (ROS) within tumor cells [1], leading to unmanageable levels of oxidative stress and consequent apoptosis. However, the cellular target of elesclomol remained unknown, as did the molecular mechanism by which it generated ROS.

Increased levels of ROS and an altered redox status have long been observed in cancer cells [2], where constitutively elevated oxidative stress and dependence on antiapoptotic ROS signaling represent potential vulnerabilities of tumors that can be exploited by small molecule drugs [3]. To test its usefulness as a therapeutic, elesclomol has been evaluated in human clinical studies [4], [5] and combined data from three randomized Phase 2 and 3 trials have demonstrated therapeutic benefit, including prolonged progression-free survival, in a subset of the patients treated [6]. Interestingly, this subset was distinguished by the prevalence of subjects with low baseline levels of serum lactate dehydrogenase (LDH). LDH level is known as a prognostic marker for outcome in cancer, but for elesclomol, it also appears also to be a predictive marker of efficacy [6]. Understanding the relationship between clinical benefit and serum LDH level at a molecular level would greatly assist the continued clinical development of the drug, including the identification of patients most likely to benefit.

**Figure 1:**
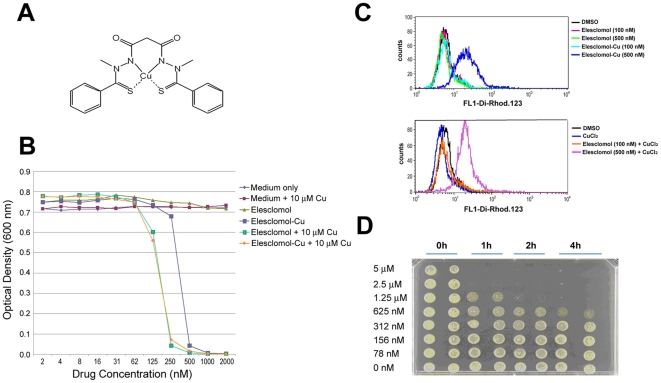
Elesclomol-induced ROS generation and cytoxicity in yeast is dependent on the presence of copper. (A) Chemical structure of the elesclomol-Cu complex. (B) The parental BY4743 yeast strain was grown in the presence of the indicated concentrations of elesclomol, preformed elesclomol-Cu, and/or copper for 21.5 h at 30°C. Absorbance at 600 nm was used to determine cell density. (C) Flow cytometric analysis measuring ROS in *S. cerevisiae*. ROS induction, as measured by Dihydrorhodamine 123 fluorescence, was only observed with preformed elesclomol-Cu complex at a concentration (500 nM) above the MIC but not below (100 nM), nor with free elesclomol at either concentration (*upper panel*). The addition of supplementary copper (via CuCl_2_) to elesclomol was sufficient to induce ROS, again only at the higher concentration (*lower panel*). (D) Elesclomol is cidal to yeast cells within an hour of treatment. Logarithmically growing cells were incubated with the indicated doses of elesclomol for 1, 2 or 4 h and then plated onto media without elesclomol. 5 µM and 22.5 µM elesclomol rendered cells unviable within 1 h, and lower doses (1.25 µM) killed cells within 4 h.

As part of the continuing effort to better understand elesclomol's cellular mechanism of action (MoA), Nagai *et al.* found that the compound binds strongly to copper (Figure 1A) and that this binding is required for its cell killing activity [7]. When administered to humans, elesclomol acquires the needed copper ions (in the form of Cu^2+^) while in the bloodstream. Copper binding changes the conformation of the drug [8], facilitates its uptake into cells, and allows it to undergo redox cycling [Cu(II) to Cu(I)] to generate ROS inside the cell [7]. In the absence of bound copper, the compound has no discernible activity.

To further refine elesclomol's MoA, we made use of a powerful comparative growth assay available in the yeast *Saccharomyces cerevisiae*. While performed in yeast, this strategy has in previous studies (particularly for cancer drugs) identified relevant mechanistic details that are indicative of the drugs' actions in humans [9], [10], [11], [12], [13]. The assay uses a set of diploid yeast strains that were systematically constructed such that each strain contains a single start-to-stop gene deletion and that the set *en toto* contains a deletion mutant for each yeast gene [14], [15]. For the ∼1100 genes that are essential for growth (in the medium employed here), those mutant strains are screened as heterozygotes. The remaining ∼4800 strains, containing mutations in non-essential genes, are screened as homozygotes. In practice, these yeast strains are grown in the presence of the drug of choice, here elesclomol, at sub-lethal doses. Deletion mutations that render the cell more sensitive to treatment serve to elucidate the MoA of the drug (for review, see [16]). For example, if the target of the drug is an essential protein in the cell, its corresponding heterozygous strain is likely to be the most growth-impaired in the collection. Similarly, analysis of the homozygous knockout strains identifies genes whose protein products are required for unabated growth when challenged with the compound. Together these screens identify the pathways associated with the drug target and the mechanisms the cell uses to counter the drug's effects [9], [17]. A critical element of this methodology is that each strain also contains a “molecular barcode” sequence in its genome that serves as a unique strain identifier [18]. Because the strains are barcoded, the entire collection of strains can be pooled and treated simultaneously with the drug. At the end of the experiment, the abundance of each barcode is quantified in parallel using Affymetrix gene chip technology such that the growth characteristics of all 6000 strains are simultaneously determined (Figure S1). The feasibility and effectiveness of this system has been demonstrated previously by screening well-characterized and novel compounds [10], [11], [12], [18], [19], [20], [21], [22].

Using this strategy, we found that elesclomol does not work through a specific cellular protein target, but rather works primarily by influencing redox reactions associated with the mitochondrial electron transport chain (ETC). This disruption leads to increased levels of ROS in the organelle and subsequently to cell death. We also show that this mechanism is conserved between yeast and human cancer cells. Taken together, these results provide a convincing explanation for the efficacy of elesclomol in low LDH patients, and support a strategy of patient stratification in future clinical work with the drug.

## Results

### Yeast are sensitive to elesclomol treatment only in the presence of copper

We initially examined whether elesclomol treatment affected the growth of yeast. Because in mammalian cells elesclomol is only active when bound to copper [7], we performed the growth analysis of the wild-type BY4743 strain (the parent of the deletion strains) in the presence of elesclomol alone, elesclomol plus varying concentrations of copper chloride (CuCl_2_), or with preformed elesclomol-Cu complex (Figure 1B). The cultures with elesclomol plus copper or elesclomol-Cu showed potent growth inhibition with a minimum inhibitory concentration (MIC) in the 250–500 nM range. Elesclomol supplemented with copper at concentrations from 10 µM to 1 mM all yielded the same MIC (data not shown). In contrast, elesclomol without added copper had no effect on yeast growth at concentrations up to 200 µM, while CuCl_2_ on its own had no effect at concentrations up to 2 mM, the latter consistent with a previous report [23].

Given the requirement for copper to induce ROS in cultured cells, we also tested whether elesclomol plus copper could induce ROS in yeast (Figure 1C). As with mammalian cells, ROS was strongly induced only in the presence of copper (either by copper supplement or as part of a preformed complex), but only at concentrations above the MIC. No ROS was observed at 100 nM elesclomol plus copper or elesclomol-Cu, consistent with ROS induction being required for growth inhibition.

Finally, we tested whether elesclomol treatment led to cell death (cidality) or simply growth arrest. Specifically, we grew wild type cells with elesclomol-Cu at varying concentrations for 1 to 4 hours with constant shaking at 30°C. Following treatment, cells were washed in fresh media and spotted onto solid growth medium without drug (Figure 1D). Elesclomol-Cu at 1.25 µM prevented colony formation after 3 h of treatment, while 2.5 µM was cidal after 1 h exposure. Taken together, these results show that elesclomol in the presence of copper, but not alone, potently kills cells after strongly inducing ROS.

### There is no cellular protein target for elesclomol

Given the conserved nature of the responses to elesclomol treatment, we reasoned that a comprehensive screen of the yeast deletion collection could reveal detailed insights into elesclomol's mechanism of action in both yeast and mammalian cells. For our assays, we used sub-MIC doses of 300 and 400 nM elesclomol-Cu, concentrations that were determined empirically to inhibit growth of the BY4743 strain by 10–20% under the conditions of the library screening. Triplicate biological replicates were then performed at both doses. An analysis of the data (the log_2_ ratio of the normalized strain signal in treatment vs. DMSO control) showed that the results were similar at both drug concentrations, so we combined all six datasets by averaging the log_2_ ratios to yield a single set of responses (Table S1). The combined values were used for all analyses presented here.

**Figure 2:**
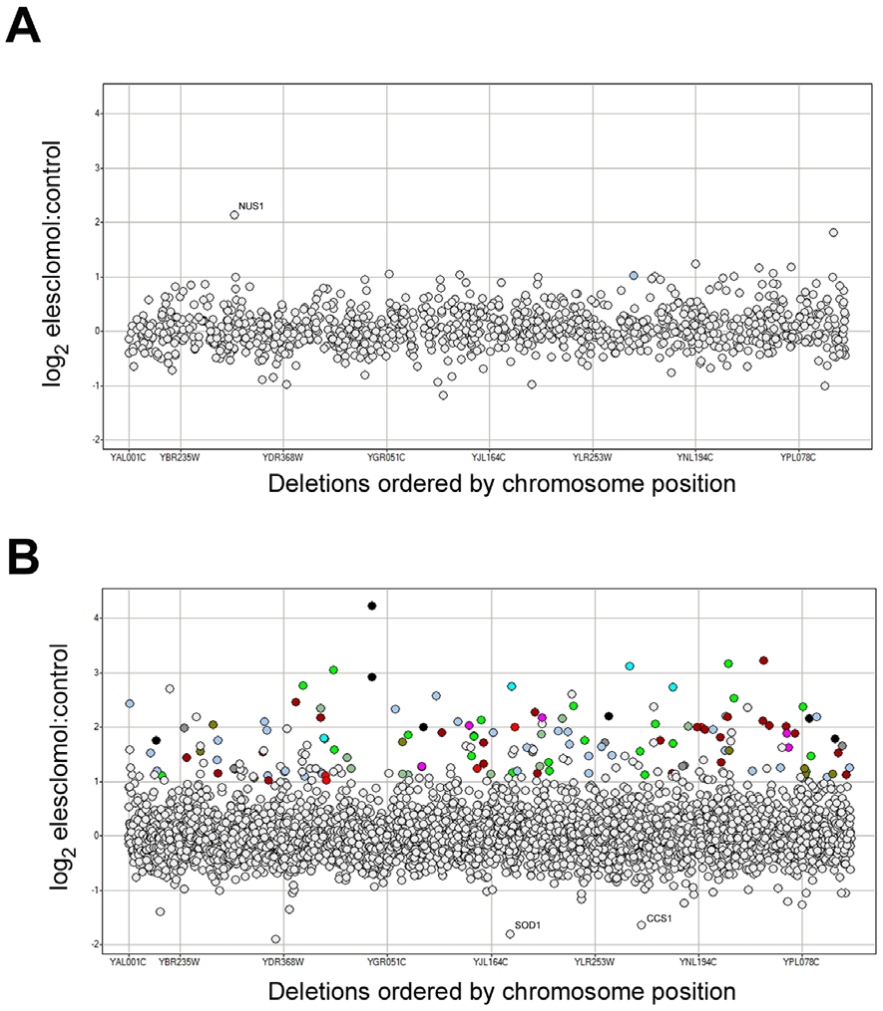
Sensitivity of *S. cerevisiae* mutant strains to elesclomol-Cu. (A) A genome-wide readout of heterozygous strain sensitivity. In the plot, the X-axis orders all genes by their systematic name (hence, chromosome position) while the Y-axis is a measure of the ‘fitness’ of the strain deleted for the indicated gene grown in sub-lethal doses of elesclomol-Cu. The value on the Y-axis corresponds to −log_2_ (ratio of normalized strain signal in treatment to DMSO control). Hence, the zero line represents equivalent growth in both conditions, while each unit above the line represents a 2-fold reduction in strain fitness. (B) Genome-wide profile of homozygous strain sensitivity. The data are presented as in *(A)*. Among the 150 most sensitive strains, the 137 having mitochondrial roles are highlighted color-coded: red (7 genes, mitochondrial genome maintenance), dark brown (8 genes, metal ion homeostasis), bright green (26 genes, mitochondrial localization), aqua (4 genes, mitochondrial, uncharacterized), blue (36 genes, ox-phos and respiration) mauve (5 genes, mitochondrial splicing), light gray (9 genes, response to stress) dark brown (26 genes, mitochondrial translation), dark gray (7 genes, mitochondrial import/export) and dark green (9 genes, mitochondrial tRNA).

As we showed previously for other drugs, if elesclomol affects cell growth by interacting or interfering with a specific protein in the cell (*i.e.*, its protein target), we expect that the heterozygous strain deleted for that gene would be highly sensitive to the drug treatment [10], [11]. In the plot shown in Figure 2A, the further up the y-axis, the more sensitive the strain, with the most sensitive strain representing the likely drug target [10]. In our experiments, the *nus1* deletion strain was the only heterozygous deletion strain that scored as sensitive (log_2_ ratio >2). *NUS1* encodes a putative prenyltransferase implicated in intracellular trafficking. However, the magnitude of the strain's sensitivity to elesclomol was modest compared to other drug-target combinations we have analyzed in the past [12], [24]. Moreover, the *nus1* heterozygote manifests a general sensitivity to a variety of compounds and conditions [12], suggesting that its sensitivity to elesclomol-Cu is not target related. Taken together, these data support the idea that yeast lack a specific protein target through which elesclomol exerts its cytotoxic activity.

### Elesclomol targets electron transport activity in the mitochondrion

In contrast, the sensitive strains among the homozygous deletions formed a biologically coherent set, consistent with elesclomol interacting with a specific target *pathway* (Figure 2B). Nearly all of the 48 strains with a log_2_ ratio >2 have a role in mitochondrial function (Table S1). At a less stringent log_2_ ratio cut-off of 1.1, ∼80% of the 190 genes are involved in mitochondrial activities. Closer inspection of these 190 reveals several interesting classes of genes. Notably, genes that are involved in diverse functions of electron transport, including structural components of the ETC (*PET309, COB1, SHY1, COQ10, YER077C, COX12, COX9, QCR2, CYT1, BCS1, COQ9, QCR7*) or involvement in the translation, modification or assembly of cytochrome components (*OXA1, CBT1, COX20, PET54, MNE1, CYT2, COQ9, COX16, CBP2, PET117, COX18*) showed sensitivity. In addition, 7 structural components of the F1F0 ATPase were sensitive as deletion alleles (*ATP1,4,7,10,11,12,17*). Several genes that comprise the mitochondrial ribosome or are directly involved in translation were identified, including: *PET123, MRPL28,23,17,31,51,27,11,38,33 RSM22,19, IFM1, MTG2, MRPS16,17*, and *MEF2* as well as 7 of the 24 mitochondrial tRNA genes (*MSM1, MSE1, MSY1, MSD1, MSF1, HER2, TPT1*). The loss of these components would likely impair the production of the mitochondrially-encoded components of the ETC. Genes involved in copper-related functions were also identified, including *COX23*, an inner mitochondrial membrane required for copper homeostasis and cytochrome oxidase expression; *COX11* which is required for delivery of copper to the Cox1p subunit of cytochrome oxidase; *COX 17*, a mitochondrial copper metallochaperone; and *CUP2*, a nuclear transcription factor that is activated in the presence of copper. Finally, genes involved in the oxidative stress response were also sensitive as deletion strains, including several whose proteins are located within mitochondria: *SOD2* (the mitochondrial superoxide dismutase), *POS5*, *MGM101*, *PRE6*, *UTH1*, and *FMP46*; as well as several reported to be present in the cytoplasm: *APD1*, *OCA1*, *RIM11*, *CNB1*, *YFR039C*, *HSP150* and *CCH1*. Interestingly, deletion of *SOD1*, the cytoplasmic superoxide dismutase, was not sensitizing, but rather conferred a degree of resistance (see discussion below).

**Figure 3:**
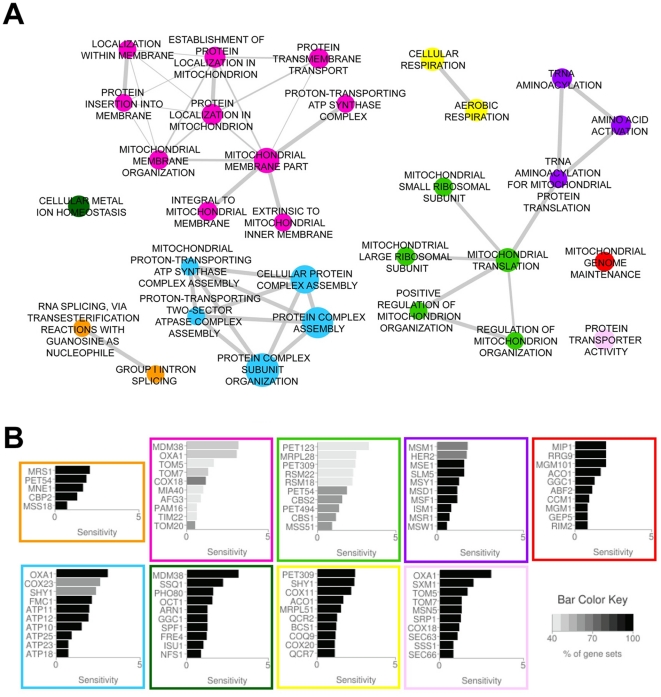
Biological processes and protein complexes associated with sensitivity to elesclomol. (A) Each node represents a significantly enriched biological process/protein complex in the elesclomol chemogenomic profile as determined by GSEA. The size of a node corresponds to the number of genes annotated to the functional category. The width of an edge corresponds to the level of gene overlap between two interconnected categories (i.e. gene sets). Edges are not shown where the overlap coefficient is less than 0.5 (see Materials and Methods). The color of a node shows the cluster membership where clustering is based on the level of overlap between categories. (B) Each bar plot corresponds to the cluster indicated by its border color, and shows the individual sensitivity scores (X-axis) of the genes that contributed to the functional enrichments of the cluster. For clusters with more than 10 genes contributing to the enrichments, only the top 10 associated with the most categories are shown. The percentage of times the particular genes occur in that gene set is indicated by a color key, with black indicating the gene is present every time that function appears enriched, graded to white for those genes in the leading edge that appear less frequently within the gene set.

To examine the yeast data more systematically, the entire dataset was analyzed by gene-set enrichment analysis (GSEA) [25]. GSEA provides an algorithmic tool to identify pathways and processes whose components are over-represented among the sensitive strains. This analysis evaluates all processes of the cell, and the only “supergroups” (major nodes) identified as significantly enriched are depicted in Figure 3A. The supergroup clusters in the figure highlight the connections between enriched functions within its biological category (with the connection strength indicated by the width of the lines connecting the functions). For each supergroup, the top 10 genes (or fewer in cases where fewer than 10 genes comprise that function) of the core gene set that is responsible for the enrichment score are shown in the histograms (Figure 3B) (see legend for details). The enriched clusters include: respiration complexes and respiration, mitochondrial membrane localization, mitochondrial tRNAs, mitochondrial genome maintenance, transport into/out of the mitochondria, splicing of mitochondrial transcripts, and metal ion homeostasis (Figure 3). Given that this analysis encompasses all biological processes and pathways described for the cell, it is striking that the only ones identified as significantly over-represented in our dataset involve mitochondrial processes or copper-related activities.

### Elesclomol works by a distinct mechanism of action

The cellular response to elesclomol identified by the subset of sensitive strains provides a unique fingerprint of the system-level response to the drug. To quantify this observation, we compared the elesclomol profile to over 3,000 drug profiles (including 300 FDA approved drugs; [26]) in our database (publicly available at (http://chemogenomics.med.utoronto.ca/hiplab/fitdb.php) and confirmed that the elesclomol profile was unique. In fact the most correlated profile showed only a modest similarity, r^2^ = 0.55. These heretofore uncharacterized compounds were derived from a synthetic compound library from ChemDiv Laboratories (San Diego, CA; compound IDs: 0352-0636, 0141-0289, 0269-0018). In contrast to the elesclomol profile, these 3 compounds show enrichment for genes primarily involved in mitochondrial protein synthesis (see http://chemogenomics.med.utoronto.ca/supplemental/elesclomol/). These data strongly suggest that elesclomol acts by a novel mechanism not shared by any previously tested compound.

Notably, we previously examined the profile produced by overloading the cell with copper, *i.e.*, growth in the presence of 10 mM CuCl_2_ [27]. The only overlap in the most sensitive strains from this treatment and that produced by elesclomol-Cu was the hypersensitivity of the *cup2* homozygous deletion strain. This rather limited overlap presumably reflects the fact that copper overload in yeast is only seen at concentrations 25,000 fold greater than that used here for elesclomol-Cu (10 mM vs. 400 nM). This strongly suggests that the cellular activity of the elesclomol-Cu complex is entirely distinct from that produced solely by copper toxicity.

### Elesclomol interacts similarly with the ETC in human cells

**Figure 4:**
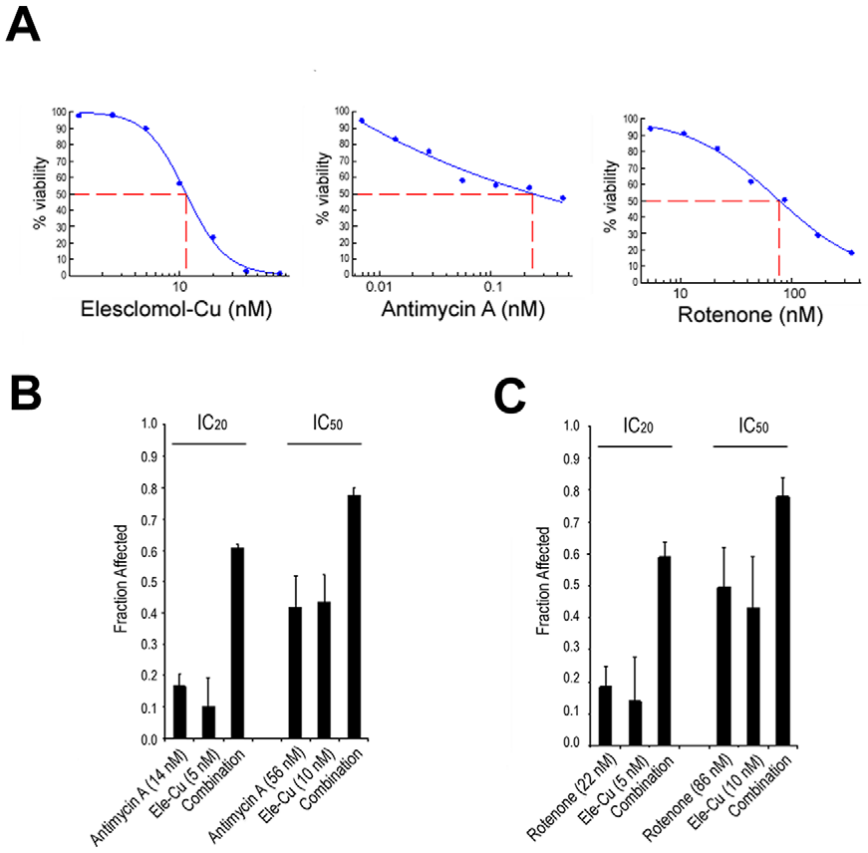
Combinations of elesclomol-Cu and ETC complex inhibitors in melanoma cells. (A) Single agent viability assays in Hs249T human melanoma cells using graded concentrations of elesclomol-Cu, antimycin A or rotenone for 72 h. The IC_50_ values obtained were 11 nM, 240 nM and 77 nM, respectively. Combination treatment of elesclomol-Cu with antimycin A (B) or rotenone (C) at IC_20_ or IC_50_ doses resulted in significantly enhanced cytotoxicity, showing that direct modulation of the ETC and mitochondrial respiration in mammalian cells enhances the cellular activity of elesclomol.

Among our sensitive yeast strains, combinations of elesclomol with specific gene deletion mutations led to greater growth impairment than would result from either insult alone. A similar approach is often undertaken with drug characterization in mammalian cells, whereby a second drug is used to examine whether co-treatment leads to synergistic effects on activity. The yeast data identified mitochondrial activities, and the ETC in particular, as processes affected by elesclomol. To determine whether this requirement for mitochondrial function and an intact ETC is conserved from yeast to human, we tested elesclomol-Cu in combination with the known ETC inhibitors antimycin A (a complex III inhibitor) or rotenone (a complex I inhibitor) in human melanoma cells (Hs294T). As single agents, both ETC inhibitors were cytotoxic, although their dose response curves were broad in comparison to the steep curve exhibited by elesclomol-Cu (Figure 4A). Concurrent administration of IC_20_ doses of elesclomol-Cu with antimycin A or rotenone resulted in substantial increases in cell death for both combination pairs and combinatorial benefit was also seen at IC_50_ doses (Figure 4B, 4C). A more extensive set of combinations, analyzed by the Chou-Talalay method based on the median effect equation [28], also showed substantial enhancement of activity with either drug upon co-administration with elesclomol (Figure S2). Although a precise quantitation of synergy is confounded by the differences in the degrees of dose response (as reflected in the shapes of the dose response curves), these results show that direct modulation of the ETC and mitochondrial respiration in human cells enhances the cytotoxic activity of elesclomol.

### Human cells lacking ETC activities are insensitive to elesclomol

**Figure 5:**
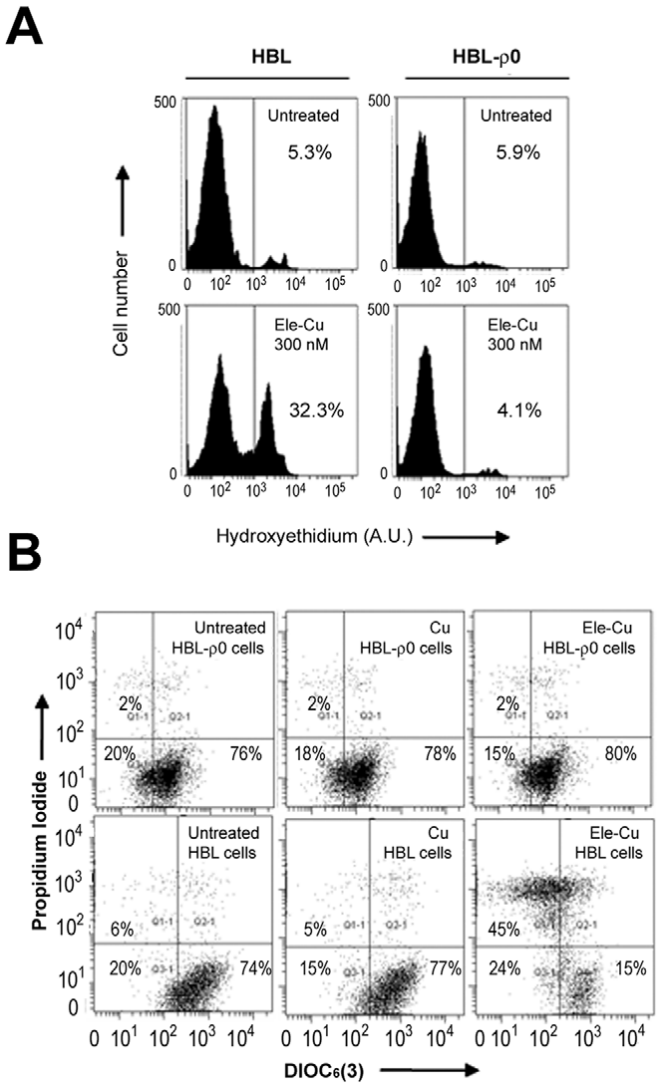
Mitochondrial DNA depleted (ρ0) melanoma cells are resistant to ROS generation and cell death upon elesclomol-Cu exposure. (A) HBL and HBL-ρ0 cells were treated for 12 h with elesclomol-Cu (300 nM). At the end of the incubation, cells were stained with hydroxyethidium to assess ROS levels by flow cytometry. Data are representative of three independent experiments. (B) HBL and HBL-ρ0 cells were treated with 300 nM elesclomol-Cu or Cu for 24 h and cell death determined using propidium iodide staining and flow cytometry.

Further evidence of a conserved MoA is provided by experiments using human HBL melanoma cells either containing their mitochondrial DNA (parent cells) or devoid of it (HBL-ρ0) [29]. Without the mitochondrial DNA, HBL-ρ0 cells cannot perform electron transport or oxidative phosphorylation, although other mitochondrial functions are maintained. HBL and HBL-ρ0 cells were treated with 300 nM elesclomol-Cu for 12 h and superoxide generation determined using a fluorescent probe (hydroxyethidium) for ROS generation. As shown in Figure 5A, ROS was readily observed in the parental cells but not in the deficient HBL-ρ0 line, indicating that elesclomol-Cu requires a functioning ETC to induce ROS. Similarly, elesclomol-Cu potently induced mitochondria-mediated cell death by 24 h in the HBL cells but not in the HBL-ρ0 cells (Figure 5B). Copper treatment alone had no effect under any of these conditions. Taken together, these findings strongly suggest that elesclomol-Cu-induced ROS generation directly translates to apoptotic effects, with both these activities dependent on the integrity of the mitochondrial ETC.

## Discussion

In a previous study on elesclomol's mechanism of action [1], the drug was shown to accomplish its cancer killing activity via the induction of untenable levels of intracellular ROS followed by apoptosis. The cellular mechanism and target by which this occurred, however, remained unknown. In those studies, mitochondrial involvement was specifically ruled out on the basis of using isolated mitochondria. However these experiments were performed in the absence of copper rendering them uninformative. In this study, with the importance of copper newly realized [7], we have used an *in vivo* yeast system and additional *in vitro* human cell studies to identify the mitochondrion as the source of elesclomol-induced ROS and strongly implicate the process of the electron transport as the “target” of the drug.

As we have shown for other cancer drugs [9], [10], [12], [17], the approach of using the yeast deletion collection yielded an accurate indication of elesclomol's mechanism of action in mammalian cancer cells. This is predicated on the similarities of the cellular responses in these different eukaryotic cells, which we found to be the case. Our results showed that both yeast and human cells require copper for elesclomol activity, induce ROS to high levels when sufficient drug is present, and succumb to cell death upon relatively short elesclomol treatment. We also reveal the importance of an active ETC in both systems.

Our data indicate the lack of a unique protein “target” of elesclomol. While the analysis of the heterozygous deletions identified a single sensitive strain, *nus1*, its sensitivity was modest compared to other drug-target strain combinations we have analyzed in the past [12], [24], and it is therefore unlikely that the interaction of elesclomol and NUS1p, if any, is responsible for the primary cytotoxic activity of elesclomol in the cell.

In contrast, the analysis of the homozygous deletion set identified a robust and biologically coherent set of activities associated with mitochondrial activities. Both manual and computational (GSEA) analyses identified overlapping classes of genes involved in various elements of electron transport, mitochondrial translation (including mitochondrial ribosome subunits, translation factors, tRNAs, and mRNA splicing enzymes), mitochondrial copper availability and homeostasis, and genes involved in stress responses, particularly oxidative stress. Importantly, equally sensitizing mutations were found distributed throughout the ETC or its associated processes. Sensitive strains containing mutations affecting individual subunits of the various ETC complexes were identified, as were components required for the modification or assembly of the complexes. Given that each of the complexes contain numerous subunits, it is likely that most of these mutations would not completely abrogate ETC function, but more likely, only partially interfere with its activity. A similar argument can be made for the sensitizing mutations affecting mitochondrial translation, which produces a minority of the proteins required for ETC function. Individually, these mutations would likely only have a small effect on the overall translation capability in the organelle, again perturbing but not eliminating ETC activity. Thus, it appears that modulating the electron flow at any of numerous points along the ETC can lead to enhanced elesclomol impact on the cell and therefore, we conclude that it is the disruption of the process of electron flow down the ETC, rather than disruption of a particular protein or activity, that is of relevance.

This effect on cell viability via ETC disruption appears to operate similarly in human cells. In the data presented in Figure 4, we used a second drug in combination with elesclomol rather that a potentially sensitizing gene disruption and obtained analogous results. Co-treatment of melanoma cells with elesclomol-Cu and either of two ETC inhibitors enhanced cytotoxicity. Again, the modulation of the process, rather than the inhibition of a particular protein, seems paramount for increased elesclomol activity.

The primary cytotoxic effect of elesclomol appears to be confined to the mitochondria and not to involve a cytoplasmic component. For example, none of the genes normally involved in the response to cytoplasmic oxidative stress were identified in the screen. This includes the *YAP1* gene, which encodes the transcription factor that is the primary responder to oxidative stress in the cytoplasm and drives the up-regulation of a battery of stress response genes. Deletion of another prominent cytoplasmic stress response protein, superoxide dismutase 1 (SOD1p), actually provided slight resistance to the elesclomol treatment. Deletion of the copper chaperone protein CCS1p, required for SOD1p activity, also provided mild resistance, thereby confirming the result. In striking contrast, deletion of *SOD2*, the mitochondrial superoxide dismutase, was highly sensitizing.

Elesclomol is thought to kill cells by inducing ROS to levels from which the cell cannot recover. Given that the principal site of action for elesclomol is likely the respiring mitochondrion, we undertook experiments utilizing human cells lacking their mitochondrial genomic DNA to examine ROS production and cytotoxicity of the drug in cells that cannot undergo oxidative phosphorylation. The human mitochondrial genome encodes 13 proteins, all of which are subunits of the various ETC complexes. The absence of all 13 subunits, as is the case in the melanoma cells used here (HBL-ρ0), ablates ETC function. These cells, when treated with elesclomol-Cu, failed to induce ROS or apoptosis. The parental cell line, with its ETC functions intact, responded with potent ROS induction and cell death. Thus, elesclomol-mediated ROS production and apoptosis are tightly linked, confirming previous analyses [1]. With the current studies, we can now propose the origin of this cascade is in the mitochondrion.

The mitochondrion is the major site for ROS production in normal cells as well. Complexes I and III are prone to electron leakage, leading to the production of highly toxic superoxide or hydroxyl radicals in the vicinity of the ETC [30]. Under most conditions these free radicals are kept in check by the anti-oxidant systems in the organelle. However, this basal level of electron leakage can be amplified by inhibitors of electron chain complexes, such as rotenone (complex I), antimycin A (complex III) or cyanide (complex IV), leading to decreased viability [31]. Similarly, the impact of elesclomol-Cu appears to overwhelm the oxidative stress response systems, allowing cytotoxic levels of ROS to accumulate. When the ETC inhibitors and elesclomol are used together during treatment, a degree of synergy is observed indicating that the combination of these drugs augments their effects in the cell as compared to monotherapy.

How might elesclomol-Cu instigate this lethal increase of ROS via its interaction with the ETC? A major clue comes from elesclomol's requirement for copper for its activity. Copper binds to elesclomol in the Cu(II) state. In the cell, elesclomol-Cu can undergo a redox reaction with copper being reduced to the Cu(I) state. By itself, this reaction could produce free radicals by a Fenton reaction. The redox potential for this reaction is −330 mV [7] and this potential appears critical for elesclomol activity. Analysis of analogs with similar structures but with different potentials has shown that only those compounds with potentials similar to elesclomol-Cu are cytotoxic [7]. Very interestingly, this potential is well aligned with the potential drops along the ETC [32].

Considering all of these features, there appears to be at least three major avenues by which elesclomol-Cu could lead to heightened levels of ROS. The drug could generate ROS on its own via its copper-based redox chemistry (perhaps using electrons or redox potential “stolen” from the ETC). Alternatively, the drug could interfere with the electron flow along the ETC, leading to elevated levels of electron leakage and free radical formation normally seen in cells, but here at levels that overwhelm the cell's defense systems. Finally, elesclomol-Cu could specifically interfere with copper-requiring events associated with ETC function. Some of the complexes are comprised of subunit proteins that require Cu for their activity and their assembly depends on specific copper chaperone proteins. Elesclomol could compete for or interfere with these processes, thereby impacting electron flow down the chain. These mechanisms are not mutually exclusive and, in fact, more than one may come into play sequentially: the initial impact of elesclomol-Cu could alter the subsequent dynamics of the ETC allowing additional mechanisms to take place that ultimately result in apoptosis. Whichever mechanism is used, we expect that the match of the redox potentials within the ETC to that of elesclomol-Cu is an important driving force.

The ability of elesclomol treatment to quickly lead to cell death, and not just cell arrest, is an important feature of the drug. Drugs that cause cidality are relatively uncommon in yeast, with fewer than 10% of 10,000 drugs that inhibit growth inducing cidality [26]. Both yeast and human cells exposed to elesclomol-Cu for a few hours or less (Figures 1 and 5) are destined to die. The ability to kill a cell exposed briefly to the drug is a valuable property for an anticancer agent.

Finally, the improved mechanistic understanding of elesclomol's activity provided by this report has important implications for its therapeutic application in oncology. Specifically, lactate dehydrogenase (LDH) has been identified as a potential biomarker predictive of response in the clinical evaluation of elesclomol. In a Phase 3 trial of elesclomol in combination with paclitaxel, the primary endpoint of progression free survival was achieved in metastatic melanoma patients exhibiting low and normal LDH levels in their bloodstream, with a significant improvement in median progression free survival time. Conversely there was no benefit in the elevated LDH population [6]. High serum levels of LDH are thought to reflect a tumor burden with increased reliance on glycolysis for its metabolic needs [33], [34]. Conversely, patients with lower LDH levels should have tumor burdens that are more reliant on oxidative phosphorylation, a situation we have shown here to be more sensitive to elesclomol treatment. Thus, the insights established here by our studies on yeast and human cells provide critical understanding into the clinical activity of the drug. It also offers a compelling rationale for a biomarker-based prioritization of patients likely to respond to elesclomol treatment.

## Materials and Methods

### Reagents

Elesclomol and elesclomol-copper complexes were synthesized at Synta Pharmaceuticals Corporation. Copper chloride, antimycin A and rotenone were all purchased from Sigma-Aldrich (St. Louis, MO).

### Yeast Strains

All strains used in this study are diploid and congenic with the reference strain BY4743 (MATa/α his3Δ1/his3Δ1 leu2Δ0/leu2Δ0 lys2Δ0/LYS2 MET15/met15Δ0 ura3Δ0/ura3Δ0).

### Minimum Inhibitory Concentration (MIC) and Cidality Determination

MIC determination was performed as previously described [9]. To test for cidality, wild type yeast (BY4743) were inoculated into YPD at an OD600 of 0.5 such that they were in the mid-log phase of growth. 100 µl of cells in media were aliquoted into wells of a 96 well plate, and a titration of elesclomol-Cu was added at a final concentration ranging from 5 nM to 5 µM in DMSO or with 2% DMSO to serve as a vehicle control. Cells were removed at hourly intervals using a pin tool to transfer 5 µl of cells in media onto agar dishes without drug. This transfer effectively dilutes the drug below inhibitory concentrations. Plated cells were incubated at 30°C for 48 h and photographed. In this assay each single viable cell transferred is able to form a visible colony. Concentrations and doses that produced no viable colonies after 48 h were scored as cidal.

### Deletion Pool Growth and Chip Experiments

Screens were performed essentially as described by Ericson *et al.* [35]. The BY4743 strain was used to determine the dose of compound that resulted in 15% growth inhibition. Cells were inoculated at an OD600 of 0.0625 in serial dilutions of drug and grown in a Tecan GENios microplate reader (Tecan Systems Inc., San Jose, CA) at 30°C with orbital shaking. Optical density measurements (OD600) were taken every 15 minutes until the cultures were saturated, and the doubling time (D) was calculated as described [19], [36].

For genome-wide fitness profiles, ∼4800 homozygous deletion strains and ∼1200 essential heterozygous deletion strains were assayed as previously reported [35] prior to genomic DNA extraction. 200 ng of genomic DNA were added to 2 separate PCR reactions, one each with primers designed to amplify all UPTAGs or all DOWNTAGS. One primer in each reaction was biotinylated such that it could be detected following hybridization to the chips using streptavidin-phycoerythrin. Intensity values for the probes on the chip were extracted using the GeneChip Operating Software (Affymetrix). Quantile normalization, outlier omission, and fitness defect ratio calculations were performed as previously described [19]. The larger the ratio, the more depleted (sensitive) is the strain as compared to control condition without the drug.

### Analysis of Elesclomol Sensitivity in Yeast Deletion Mutants

The homozygous and heterozygous deletion strains were treated *en masse* with 300 or 400 nM elesclomol-Cu, producing ∼15% growth inhibition. Each screen was performed in triplicate at both drug concentrations. Because all six sets of data yielded similar results, the log_2_ ratios from all of them were averaged. This averaged data were used for subsequent analysis.

### Gene-set Enrichment Analysis (GSEA)

Strains in the chemogenomic profile of elesclomol were mapped to genes using chromosomal feature data downloaded from the *Saccharomyces* Genome Database (http://www.yeastgenome.org) and the resulting profile was analyzed by GSEA v2.07 in pre-rank mode (Java implementation). All default parameters were used except that the minimum and maximum gene set sizes were restricted to 5 and 300, respectively. Biological process and protein complex gene annotations were obtained from Gene Ontology (http://berkeleybop.org/goose). Additional protein complex annotations based on consensus across different studies were obtained from Benschop *et al.* [37]. The enrichment map was generated with the Enrichment Map Plugin v1.1 [38] developed for Cytoscape [39]. All default parameters were used. The nodes in the map were clustered with the Markov clustering algorithm [40], using the overlap coefficient computed by the plugin as the similarity metric (coefficients less than 0.5 were set to zero) and an inflation of 2. For each cluster, the leading edge genes were computed as in [25] for each member node.

### Multiple Drug Effect Analysis

Hs294T melanoma cells were purchased from American Type Culture Collection (Manassas, VA) and cultured according to standard techniques at 37°C in 5% (v/v) CO_2_ in DMEM plus 10% FBS. The half maximal inhibitory concentration (IC_50_) values for elesclomol, rotenone and antimycin A in Hs294T cells were determined using a 1.5-fold serial dilution series of each compound. For combinatorial analysis, cells were plated in triplicate for 24 h prior to the addition of drug or vehicle (0.3% DMSO) to the culture medium. Combinations of elesclomol with rotenone or antimycin A were performed concurrently based on the IC_50_ and IC_20_ values for each agent. At 72 h, metabolic activity was monitored by alamarBlue (Invitrogen, Carlsbad, CA) fluorescence (560_EX_/590_EM_ nM) with a SpectraMax microplate reader (Molecular Devices). The resulting data were used to calculate cell viability, normalized to vehicle control. Results were fit to a four parameter logistic model (XLFit, ID Business Solutions). Combinatorial effects were additionally analyzed using the Chou and Talalay Median Effect method [28] to determine combination index (CI) values.

### Cytofluorometric analyses of ρ0 Melanoma Cell Lines

HBL and respiration-deficient HBL-ρ0 melanoma cells [29] were cultured at 37°C in a humidified atmosphere containing 5% CO_2_ in RPMI 1640 medium supplemented with 10% FCS, 2 mM L-glutamine, 100 units/ml penicillin, 100 µg/ml streptomycin and 2 g/l glucose. HBL-ρ0 cells were routinely maintained in medium containing ethidium bromide, although the dye was removed 48 h before the cells were used in experiments.

For ROS assessment, HBL and HBL-ρ0 cells were treated with elesclomol-Cu (300 nM) for 12 h and superoxide production within the mitochondria was determined using 1 µM hydroxyethidium, as previously described [41]. Oxidized ethidium fluorescence was analyzed on a FACS Canto cytofluorometer equipped with the FACS Diva software 6.1 (Becton Dickinson, San Diego, CA). For viability assays, HBL and HBL-ρ0 cells were incubated with 300 nM elesclomol-Cu or Cu for 24 h. Cell death was determined using propidium iodide (5 µg/ml in PBS) staining and FACS analysis. Inner mitochondrial transmembrane potential was measured by incubating cells (25×10^4^/ml) with 40 nM DIOC_6_(3) for 15 minutes at 37°C immediately prior to flow cytometry.

## Supporting Information

Figure S1 **Competitive growth assay and complementary whole-genome population genomics in** ***S. cerevisiae*** **.** For fitness profiling, deletion strains (heterozygotes and homozygotes) are first pooled at approximately equal abundance. The pool is then grown competitively in the presence or absence of drug, its genomic DNA recovered en masse, and the DNA barcodes PCR-amplified in two reactions (to amplify separately the UPTAG and DOWNTAG barcodes from each strain). The PCR products are hybridized to a tag array that contains the barcode complements (5 copies of each are present on each chip), and tag intensity is used to determine changes in the amount of each strain present. Strains with deletions in genes that are important for survival during drug treatment will be underrepresented in the treated sample compared to the control. These genes are either the target of the drug (if the gene is essential) or components of critical pathways influencing the activity of the drug (if the genes are non-essential). Figure modified from Fleming *et al.* [9].
(TIF)

Figure S2 **Combinatorial activity of elesclomol-Cu with ETC inhibitors in Hs924T melanoma cells.** Normalized isobolograms for the concurrent treatment of elesclomol-Cu with antimycin A (*left panel*) or rotenone (*right panel*) in Hs924T melanoma cells using non-constant ratios. Combination Index (CI) values were calculated using Median Effect analysis [28]. A point in the isobologram represents the effect of a combinatorial drug treatment. The further a point lays from the additive line, the stronger the (synergistic or antagonistic) impact of the combination. The respective drug concentrations (in nM) for each combination shown are as follows. For the elesclomol-Cu/antimycin A combination: point 1, 5/14; point 2, 5/28; point 3, 5/56; point 4, 10/14; point 5, 10/28; point 6, 10/56. For the elesclomol-Cu/rotenone combination: point 1, 5/21.5; point 2, 5/43; point 3, 5/86; point 4, 10/21.5; point 5, 10/43; point 6, 10/86.
(TIF)

Table S1 **Growth responses of individual mutant strains to treatment with elesclomol.** Sub-MIC doses of 300 or 400 nM elesclomol-Cu were used to treat *en masse* the collection of deletion strains as described in the Materials and Methods. Three biological replicates were performed at each dose. An analysis of the fitness data (the log_2_ ratio of the normalized strain signal in treatment vs. DMSO control) showed that the results were similar for both drug concentrations, so all six datasets were combined by averaging the log_2_ ratios to yield the single set of responses reported in the Table. These averaged fitness values were used for all analyses presented in the paper.
(XLSX)
