# Supplementary figures and images for: Mitochondrial Electron Transport Is the Cellular Target of the Oncology Drug Elesclomol
Source: PLoS One. 2012 Jan 11;7(1):e29798. doi: 10.1371/journal.pone.0029798 (PMC3256171; doi:10.1371/journal.pone.0029798)

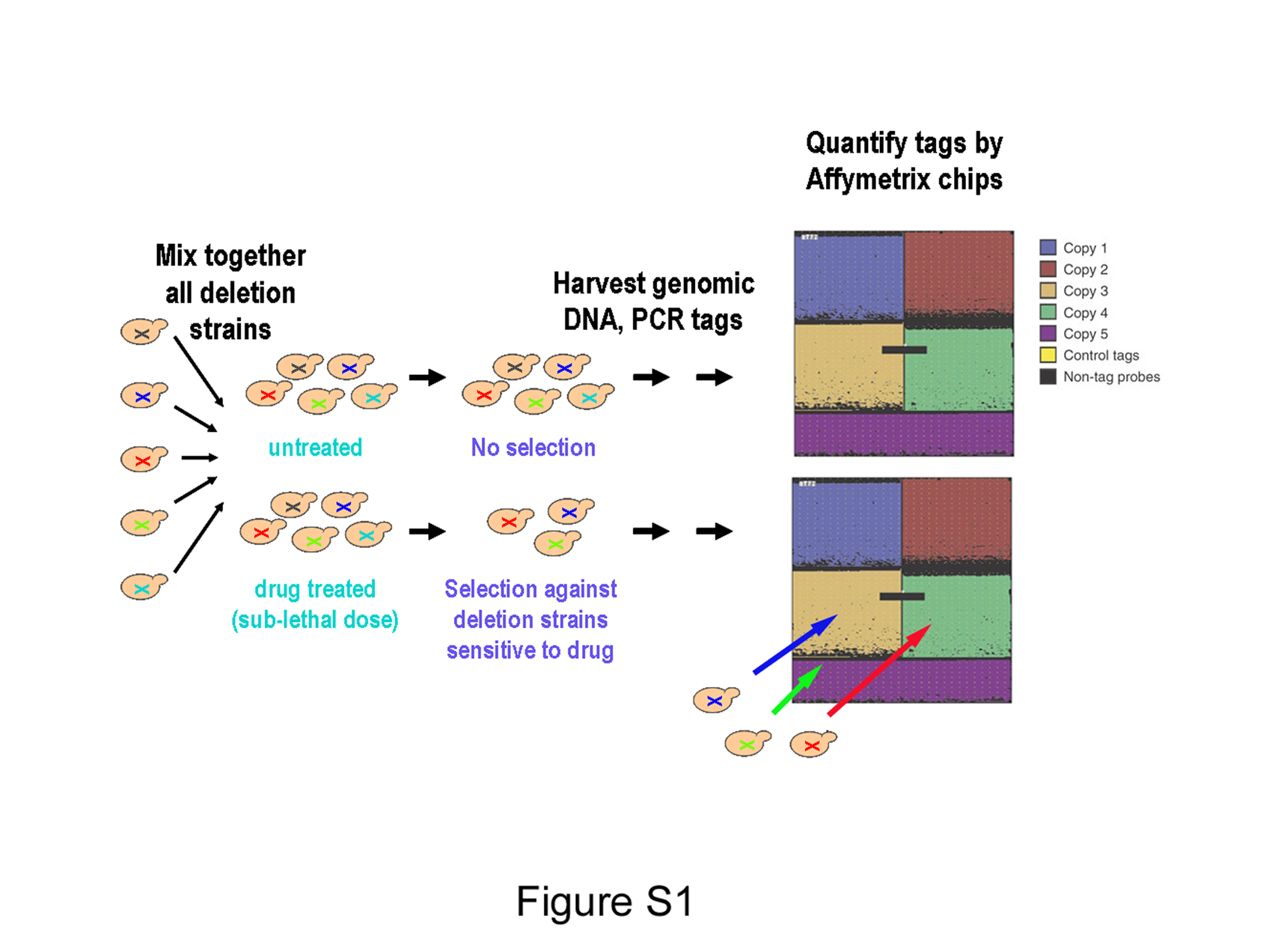

Supplement: Figure S1 — Competitive growth assay and complementary whole-genome population genomics in S. cerevisiae . For fitness profiling, deletion strains (heterozygotes and homozygotes) are first pooled at approximately equal abundance. The pool is then grown competitively in the presence or absence of drug, its genomic DNA recovered en masse, and the DNA barcodes PCR-amplified in two reactions (to amplify separately the UPTAG and DOWNTAG barcodes from each strain). The PCR products are hybridized to a tag array that contains the barcode complements (5 copies of each are present on each chip), and tag intensity is used to determine changes in the amount of each strain present. Strains with deletions in genes that are important for survival during drug treatment will be underrepresented in the treated sample compared to the control. These genes are either the target of the drug (if the gene is essential) or components of critical pathways influencing the activity of the drug (if the genes are non-essential). Figure modified from Fleming et al. [9]. (TIF) [file pone.0029798.s001.tif]

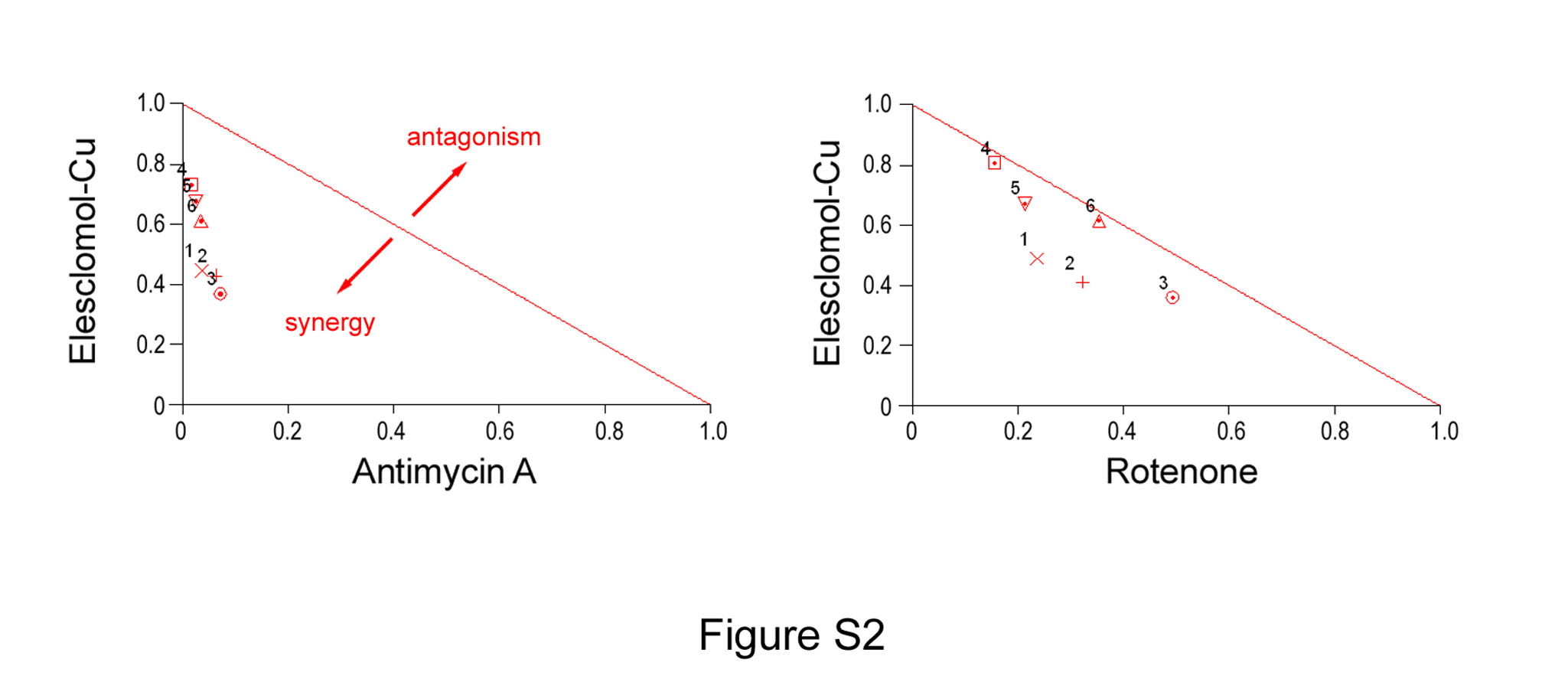

Supplement: Figure S2 — Combinatorial activity of elesclomol-Cu with ETC inhibitors in Hs924T melanoma cells. Normalized isobolograms for the concurrent treatment of elesclomol-Cu with antimycin A (left panel) or rotenone (right panel) in Hs924T melanoma cells using non-constant ratios. Combination Index (CI) values were calculated using Median Effect analysis [28]. A point in the isobologram represents the effect of a combinatorial drug treatment. The further a point lays from the additive line, the stronger the (synergistic or antagonistic) impact of the combination. The respective drug concentrations (in nM) for each combination shown are as follows. For the elesclomol-Cu/antimycin A combination: point 1, 5/14; point 2, 5/28; point 3, 5/56; point 4, 10/14; point 5, 10/28; point 6, 10/56. For the elesclomol-Cu/rotenone combination: point 1, 5/21.5; point 2, 5/43; point 3, 5/86; point 4, 10/21.5; point 5, 10/43; point 6, 10/86. (TIF) [file pone.0029798.s002.tif]
